# Successful pulmonary administration of activated recombinant factor VII in diffuse alveolar hemorrhage

**DOI:** 10.1186/cc5132

**Published:** 2006-12-21

**Authors:** Lars Heslet, Jorn Dalsgaard Nielsen, Marcel Levi, Henrik Sengeløv, Pär I Johansson

**Affiliations:** 1 Department of Intensive Care ITA 4131, University Hospital of Copenhagen, Rigshospitalet, Blegdamsvej 9, DK 2100 Denmark; 2 Department of Clinical Biochemistry, Gentofte University Hospital, Niels Andersens Vej 65, DK 2900 Hellerup, Denmark; 3 Department of Internal Medicine/Vascular Medicine, Amsterdam Medical Center, De Boelelaan 1117, 1081 HV, Amsterdam, The Netherlands; 4 Department of Hematology H, University Hospital of Copenhagen, Rigshospitalet, Blegdamsvej 9, DK 2100 Denmark; 5 Department of Clinical Immunology, University Hospital of Copenhagen, Rigshospitalet, Blegdamsvej 9, DK 2100 Denmark

## Abstract

**Introduction:**

Diffuse alveolar hemorrhage (DAH) is a serious pulmonary complication seen in patients with autoimmune disorders and patients treated with chemotherapy or after hematopoietic stem cell transplantation. The clinical management of DAH is complex and the condition has a high mortality rate. Tissue factor is expressed in the lung alveoli during inflammation and therefore pulmonary administration of human recombinant activated factor VIIa (rFVIIa) could be a rational treatment option.

**Methods:**

Six patients with acute, bronchoscopically confirmed DAH from a single intensive care unit university hospital center were included in the study of acute DAH in critically ill patients. The patients were treated with intrapulmonary administration of 50 μg/kg rFVIIa in 50 ml of sodium chloride by bronchoalveolar lavage (BAL) with 25 ml in each of the main bronchi, which was repeated after 24 hours in case of treatment failure.

**Results:**

An excellent response, defined as complete and sustained hemostasis after a single dose of rFVIIa, was seen in three patients. A good response, meaning that sustained hemostasis was achieved by a repeated rFVIIa administration, was seen in the remaining three patients. In one of these patients, the BAL treatment was repeated twice; in another patient, the second dose of rFVIIa was administered by nebulizer after extubation after the initial BAL. The hemostatic effect was statistically significant (*p *= 0.031). The oxygenation capacity, as reflected by the PaO_2_/FiO_2 _(arterial oxygen pressure/inspiratory fractional oxygen content) ratio, increased significantly (*p *= 0.024) in all six patients following the local rFVIIa therapy.

**Conclusion:**

Symptomatic therapy of DAH after intrapulmonary administration of one or more doses of rFVIIa was found to have a good to excellent hemostatic effect in six consecutive patients with DAH. The intrapulmonary administration of rFVIIa seemed to have a high benefit-to-risk ratio. Larger series should confirm the safety of this approach.

## Introduction

Diffuse alveolar hemorrhage (DAH) is a serious pulmonary complication of mostly unknown etiology and pathogenesis, although injury to alveolar capillary endothelium and alveolar inflammation, resulting in the release of inflammatory cytokines, have been implicated [1,2]. The disease is seen after hematopoietic stem cell transplantation (HSCT), after chemotherapy, and in patients with autoimmune disorders [3]. The extensive pulmonary inflammation leads to abundant intra-alveolar expression of tissue factor (TF), resulting in a several-fold increase in molecular markers of thrombin generation in bronchoalveolar lavage (BAL) fluid [4]. Effective local hemostatic strategies are lacking, and mortality rates exceed 50% in those who require mechanical ventilator support [5]. We hypothesized that local administration of human recombinant activated factor VIIa (rFVIIa) might be an effective treatment option.

## Materials and methods

Six consecutive patients with pulmonary bleeding, of whom four had an inspiratory fractional oxygen content (FiO_2_) demand of 1.0, not responding to conventional therapy were studied. At our institution, treatment of pulmonary bleeding includes transfusion of fresh frozen plasma (FFP) and platelet concentrate (PC) to normalize systemic coagulation ability, endotracheal and intravenous (i.v.) administration of tranexamic acid, and if no hemostatic effect is obtained, this is followed by continuous aprotinin i.v. infusion. The diagnosis of DAH was confirmed bronchoscopically by identification of ongoing bleeding at the bronchial segmental level before treatment with rFVIIa. The dose was approximately 50 μg/kg dissolved in 50 ml of saline distributed evenly in the right and left main bronchi. In one non-intubated patient, rFVIIa was administered as an inhalant at a dose of 50 μg/kg via a jet-driven nebulizer.

Treatment efficacy of rFVIIa was graded as an excellent, good, or poor response. An 'excellent' response was defined as a complete and sustained hemostasis after a single treatment with rFVIIa. The response was graded as 'good' when repeated intrapulmonary administration of rFVIIa was required to obtain hemostasis. A 'poor' response was characterized by the lack of any effect by rFVIIa on bleeding. The hemostatic effect and the oxygenation capacity were statistically analyzed using the non-parametric tests, McNemar's test and Wilcoxon signed paired rank test, respectively. A waiver of informed consent was obtained from the Institutional Review Board.

## Results

### Patients

#### Patient 1

A 46-year-old male with chronic lymphocytic leukemia underwent allogenic non-myeloablative stem cell transplantation (HSCT). The post-transplant course was complicated by severe graft-versus-host disease (GvHD) of the skin, thrombocytopenia, and systemic cytomegalovirus (CMV) infection, causing treatment-induced renal failure that necessitated hemodialysis (Table 1).

Nine months after HSCT, he was admitted to the intensive care unit (ICU) with septic shock, which was treated with empiric antibiotics, vasoactive medication, and mechanical ventilation. Seven days after ICU admission, the FiO_2 _requirement suddenly increased to 1.0 and fresh blood was coming from the endotracheal tube. The platelet count was 10 × 10^9 ^per liter and the activated partial thromboplastin time (APTT) was 40 seconds. Despite platelet transfusion to a platelet count of more than 100 × 10^9 ^per liter and FFP administration to an APTT of less than 35 seconds (in addition to the standard treatment as described in Materials and methods), the bleeding increased in severity. rFVIIa was administered intravenously in doses of up to 120 μg/kg without hemostatic effect. Bronchoscopy revealed fresh bleeding from both lungs. rFVIIa (50 μg/kg) dissolved in saline was administered by BAL into the left and right main bronchi simultaneously with the systemic i.v. administration of 50 μg/kg rFVIIa. This resulted in an immediate cessation of the bleeding from the endotracheal tube. The arterial oxygen pressure (PaO_2_)/FiO_2 _ratio increased the subsequent day. The hemostatic effect lasted for approximately 36 hours, after which bleeding recurred. The treatment was repeated twice also, and bleeding ceased for more than 24 hours. Hereafter, the patient received rFVIIa (50 μg/kg) by BAL alone twice, and bleeding ceased for 24 to 36 hours after each administration. Complete hemostasis was obtained after the third BAL administration of rFVIIa and lasted for three months (Table 2). The patient died three months after the first rFVIIa treatment, due to respiratory and circulatory failure secondary to septic shock without evidence of active pulmonary bleeding. Postmortem examination revealed no signs of acute respiratory distress syndrome (ARDS) in the alveoli or thromboembolic complications.

#### Patient 2

A 63-year-old male with progressive neurosarcoidosis was treated with infusion of methotrexate. After the second treatment, he developed pancytopenia and septic shock and was transferred to the ICU, where he was intubated and mechanically ventilated. Fresh blood was observed from the tracheal tube, and chest x-ray showed interstitial diffuse alveolar infiltrates indicating pulmonary bleeding. Standard treatment was instituted, resulting in an increase in platelet count from 18 × 10^9 ^per liter to 88 × 10^9 ^per liter with a reduction in APTT from 45 to 36 seconds (Table 2). Multiple organ failure developed and increasing amounts of fresh blood were observed from the tube while the FiO_2 _requirement increased from 0.7 to 1.0. A single dose (20 μg) of i.v. desmopressin did not improve hemostasis. Bronchoscopy revealed ongoing bleeding from the distal bronchial tree bilaterally. A 50 μg/kg dose of rFVIIa was administered via BAL, resulting in immediate cessation of the pulmonary bleeding. Four hours after rFVIIa administration, FiO_2 _could be reduced to 0.8 and was further reduced to 0.6 the following morning (Table 2). Despite continued improvement in pulmonary function, increasing circulatory instability secondary to septic shock became evident and the patient died four days after rFVIIa treatment. No pulmonary bleeding or thromboembolic complications were found after the intrapulmonary administration of rFVIIa.

#### Patient 3

A 44-year-old male with acute myeloid leukemia (AML) developed high fever and hemoptysis 14 days after induction of a combination of chemotherapy comprising cytarabine, amsacrine, and etoposide. Chest x-ray showed bilateral infiltrations, and BAL revealed *Stenotrophomonas maltophilia*. The patient was admitted to the ICU, where mechanical ventilation was instituted with an FiO_2 _of 1.0, and was started with inotropic support and antimicrobial therapy (i.v. ceftazidime and inhalation of colistin). Pulmonary bleeding increased despite standard treatment (Table 2). Bronchoscopy revealed ongoing bleeding from the distal bronchial tree bilaterally, and rFVIIa (50 μg/kg) was administered by BAL. The bleeding ceased and the FiO_2 _was decreased to 0.45 over the next 24 hours. Over the next 12 days, the situation stabilized and improvement of the pulmonary and the circulatory functions was observed and the patient could be weaned off of the ventilator and discharged from the ICU. No pulmonary bleeding or thromboembolic complications were observed after the intrapulmonary administration of rFVIIa.

#### Patient 4

A 34-year-old woman was suspected of having Wegener's granulomatosis on the basis of eosinophilia, positive anti-neutrophil cytoplasmatic antibodies, hematuria, and proteinuria. Treatment with systemic corticosteroids was instituted, but due to respiratory distress and the presence of hemoptysis, the patient was admitted to the ICU. Chest x-ray showed interstitial subtle bilateral alveolar infiltrates indicating pulmonary bleeding. The patient was intubated and mechanically ventilated with an FiO_2 _of 1.0, and systemic antibiotic treatment was initiated in conjunction with pulse treatment with methylprednisone (1,000 mg intravenously every day for three days and then 40 mg every day). Treatment including continuous aprotinin infusion was instituted. The pulmonary bleeding ceased, FiO_2 _demand was reduced to 0.35, and antifibrinolytic treatment was discontinued. Twelve hours after discontinuation of aprotinin, fresh pulmonary bleeding again became apparent, together with an increase in FiO_2 _requirements to 1.0. The bleeding was refractory to standard treatment, including aprotinin. Bronchoscopy revealed ongoing bleeding at the segmental level, and rFVIIa (50 μg/kg) was administered by BAL, leading to immediate cessation of the bleeding; FiO_2 _was reduced to 0.3 at six hours after rFVIIa treatment, and the patient was extubated the following morning.

A biopsy from the skin showed perivascular eosinophilic infiltration indicating Churg-Strauss vasculitis, and the patient responded to the corticosteroid therapy treatment with regression of paresis and normalization of urine tests. Three days after extubation, bleeding from the lungs reoccurred together with an increase in O_2 _demand. To avoid re-intubation, rFVIIa was administered through a jet nebulizer with a prompt hemostatic effect. The aerosolized rFVIIa was repeated twice over the following 12 hours. A sustained hemostasis and a decrease in O_2 _requirements from 15 to 4 liters/minute were obtained. The further clinical course was uneventful, and the patient was discharged from the ICU three days later.

#### Patient 5

A 44-year-old HIV-positive female with chronic hemodialysis requirement, severe critical illness polyneuropathy, and enterocolitis due to *Clostridium difficile *infection, for which she received i.v. vancomycin, underwent surgery due to gastrointestinal bleeding. Postoperatively, she was transferred to the ICU, receiving mechanical ventilator support, and developed ventilator-associated pneumonia due to *Pseudomonas aeruginosa*, which was treated successfully with broad-spectrum antibiotics. In addition, a systemic CMV infection developed for which she was treated with Foscarnet, leading to stabilization over the following weeks. At 51 days after ICU admission, however, fresh bleeding occurred from the tracheotomy but did not respond to standard treatment as described previously. BAL revealed localized bleeding at the segmental level bilaterally, and rFVIIa (50 μg/kg) dissolved in 50 ml of sodium chloride was administered with 25 ml in each main bronchus. The pulmonary bleeding ceased but reappeared within 24 hours after the treatment, and rFVIIa administration by BAL was repeated. Hereafter, the bleeding ceased to occur. The patient expired due to infection and respiratory insufficiency 115 days after rFVIIa treatment, without any signs of thromboembolic complications.

#### Patient 6

A 63-year-old male with AML underwent non-myeloablative stem cell transplantation. The post-transplant course was complicated by GvHD of the skin and temporary poor graft function with pancytopenia. Six months post-transplant, the patient was transferred to the ICU due to respiratory insufficiency secondary to pulmonary infection. The patient was intubated and mechanically ventilated with an FiO_2 _of 0.45. Diagnostic BAL showed fresh blood at segmental levels bilaterally, but the focus of bleeding could not be identified. The platelet count was 35 × 10^9 ^per liter and APTT was 40 seconds, for which the patient received FFP to achieve an APTT of less than 30 seconds and PCs to achieve a platelet count of more than 80 × 10^9 ^per liter but without significant effect on the bleeding. The patient received empirical broad-spectrum antibiotics and antimycotics, resulting in a decrease of the C-reactive protein over the next five days. Twelve days after ICU admission, the pulmonary bleeding increased and the FiO_2 _demand was increased to 0.6. The patient had a normal TEG (thrombelastografic *in vitro *coagulation) profile, a platelet count of 80 × 10^9 ^per liter, and an APTT of 27 seconds, indicating a localized coagulopathy. Due to further increase in bleeding and FiO_2 _demand, a diagnostic BAL was performed, showing fresh bleeding bilaterally at segmental levels; rFVIIa at a dose of 50 μg/kg dissolved in 50 ml of saline was administered, resulting in immediate cessation of the pulmonary bleeding. The FiO_2 _was reduced to 0.35 within the next eight hours, and the patient was extubated the following morning. Three days after treatment with local pulmonary rFVIIa, the patient was discharged from the ICU without further bleeding episodes.

## Discussion

DAH is a clinical syndrome with acute onset of alveolar infiltrates and hypoxemia, yielding progressively diffuse alveolar bleeding. Clinical features include dyspnea, cough, hemoptysis, abnormal chest x-ray with bilateral alveolar infiltrates, and hypoxia usually accompanied with fever [6,7]. The treatment of DAH is empiric in as much as the condition is a life-threatening medical emergency with no specific or proven effective therapy. Treatment with high-dose steroids may be beneficial when given early [7], but overall mortality remains high; plasmapheresis has been advocated, but there is no evidence that this intervention is successful in the treatment of ongoing low-volume critical bleeding [8].

Here, we report a series of six patients of DAH verified by bronchoscopy in mechanically ventilated patients. There was no or insufficient hemostatic effect of standard therapy including transfusion of FFP and PCs, i.v. infusion of aprotinin, and tranexamic acid intravenously or via the endotracheal route (Table 1).

rFVIIa is an approved agent for the i.v. treatment of bleeding episodes in patients with hemophilia A or B with inhibiting antibodies toward factor VIII or factor IX, respectively. Factor VII initiates clot formation by its interaction with TF [9,10]. The FVIIa-TF complex activates factor X. In high doses (80 to 100 μg/kg), however, activated FVII also activates factor X in the absence of TF, probably by activation of factor X bound to the surface of activated platelets. Activated factor X activates prothrombin to thrombin, which in turn converts fibrinogen to fibrin [10].

High and repeated i.v. doses of rFVIIa have been reported to have some hemostatic effect in patients with DAH [11-13]. As described in this report, however, i.v. administration of a very high dose of rFVIIa did not induce hemostasis in our first patient with DAH. This led us to explore the effect of local pulmonary administration of rFVIIa, and the efficacy of this treatment was demonstrated in six consecutive patients with DAH of different etiologies. The rFVIIa was administered at a dose of approximately 50 μg/kg via BAL in six patients and as a nebulized aerosol on one occasion. The intervention with local intrapulmonary rFVIIa had a significant hemostatic effect (*p *= 0.031). To our knowledge, this is the first time an effective treatment of DAH using symptomatic treatment with local intrapulmonary rFVIIa has been reported.

**Figure 1:**
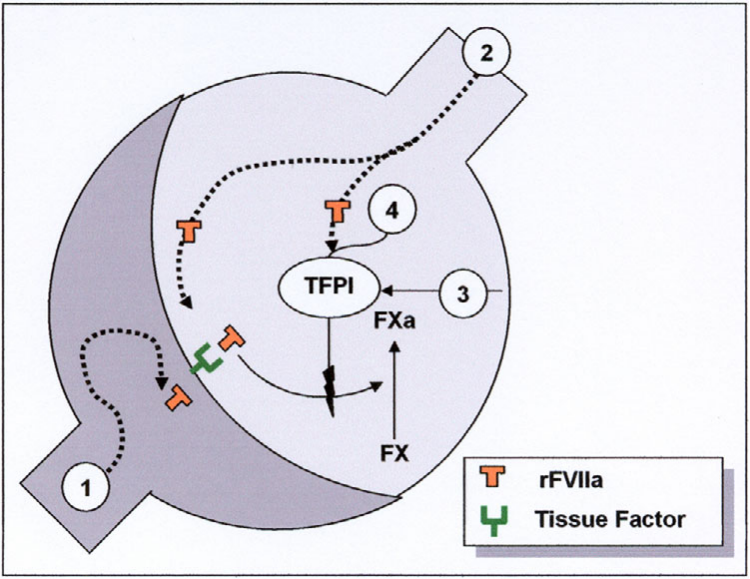
Rationale for the local mode of action of intra-alveolar human recombinant activated factor VII (rFVIIa) in diffuse alveolar hemorrhage (DAH). Intravenous rFVIIa does not reach the alveoli in a sufficient concentration (1) in contrast to the airway route (2). Alveolar tissue factor (TF)-FVIIa complex activates coagulation factor IX and X. TF and TF pathway inhibitor (TFPI) are constitutively expressed in the airspace, secondary to inflammation induced in DAH (3). TFPI counteracts the activation effect of the FVIIa-TF complex. Alveolar rFVIIa in high concentration counteracts the TFPI anticoagulation (4).

Clinical observation supports the hypothesis that pulmonary hemostasis can be induced more effectively from the alveolar side in DAH and than from the endothelial side. This viewpoint is supported by the fact that alveolar TF is demonstrated in high concentrations in inflammatory pulmonary conditions like ARDS [14], pneumonia [15], and after lipopolysaccharide challenge locally in the alveoli [16]. The mode of action of the observed alveolar hemostasis is most likely primarily explained by the TF-dependent pathway, where alveolar TF is expressed during the inflammatory phase of DAH. On the other hand, TF pathway inhibitor (TFPI) is a strong inhibitor of the local activation of factor X to Xa by the FVIIa-TF complex. In acute lung injury, TFPI produced by alveolar macrophages may be increased 20-fold [17]. Our observations indicate that intrapulmonary administration of FVIIa overrides the anticoagulant effect of TFPI (Figure 1).

**Figure 2:**
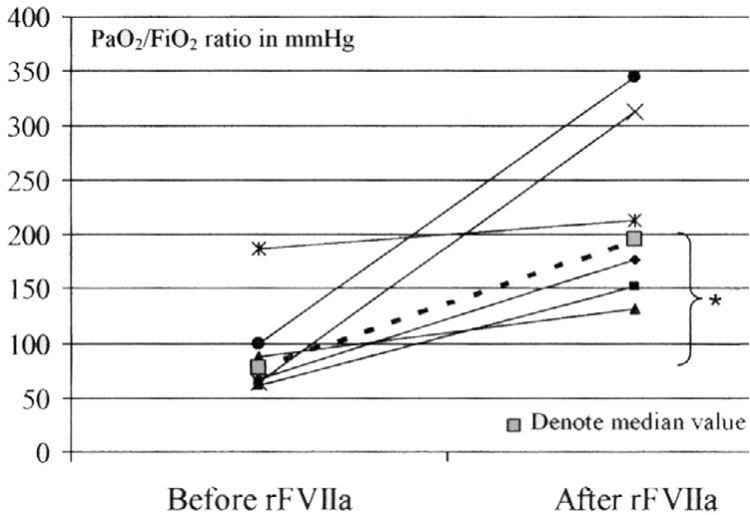
Oxygenation capacity before and after local pulmonary human recombinant activated factor VII (rFVIIa) therapy. A significant improvement in PaO_2_/FiO_2 _(arterial oxygen pressure/inspiratory fractional oxygen content) ratio was observed after the hemostatic treatment (*p = 0.024, Wilcoxon signed paired rank test).

A safety issue of the local rFVIIa treatment, however, is the possible risk of inducing widespread alveolar fibrin deposition (that is, hyaline membrane formation), which is a hallmark of ARDS. There were, however, no signs of developing ARDS in the six treated patients because the oxygenation capacity, as reflected by the PaO_2_/FiO_2 _ratio, increased significantly in the six patients after the pulmonary rFVIIa administration (Figure 2). The benefit, the hemostatic effect, was excellent in three patients and good in three patients (that is, a statistically significant treatment effect was observed in all patients) (Table 2). The benefit-to-risk ratio of local rFVIIa treatment in DAH therefore seems to be high.

## Conclusion

A new symptomatic therapy, involving intrapulmonary administration of rFVIIa, to stop the life-threatening critical bleeding in DAH is documented in six consecutive patients with DAH. It seems that pulmonary hemostasis occurs most likely from the alveolar side in DAH and to a much lesser extent from the lung vascular endothelial side, a viewpoint that is supported by the clinical observation of the patients with DAH and by the well-described pathophysiology of the lung as a hemostatic organ, with TF-dependent and TF-independent modes of action. But irrespective of the mode of action, FVIIa has a potentially high benefit-to-risk ratio when administered via the local intrapulmonary route. These findings warrant further exploration of the local pulmonary effect of rFVIIa and the safety of this novel treatment strategy in patients with DAH.

## Key messages

• DAH has a high mortality and no documented specific intervention.

• Symptomatic therapy with local intrapulmonary therapy with one or more doses of recombinant FVIIa was found to have a good to excellent hemostatic effect in patients with DAH.

• No adverse effects could be demonstrated.

• The treatment with rFVIIa seems to have a high benefit-to-risk ratio in DAH.

## Abbreviations

AML = acute myeloid leukemia; APTT = activated partial thromboplastin time; ARDS = acute respiratory distress syndrome; BAL = bronchoalveolar lavage; CMV = cytomegalovirus; DAH = diffuse alveolar hemorrhage; FFP = fresh frozen plasma; FiO_2 _= inspiratory fractional oxygen content; GvHD = graft-versus-host disease; HSCT = hematopoietic stem cell transplantation; ICU = intensive care unit; i.v. = intravenous; PaO_2 _= arterial oxygen pressure; PC = platelet concentrate; rFVIIa = human recombinant activated factor VII; TF = tissue factor; TFPI = tissue factor pathway inhibitor.

## Competing interests

LH has shares in the pharmacompany Pharmaorigin, Copenhagen, Denmark, which is holding a patent related to the local pulmonary treatment with rFVIIa, but has not received reimbursements, fees, funding, or salary from any organization relating to the content or the preparation of the manuscript. LH declares that he has no other competing interests. JDN, ML, HS, and PIJ declare that they have no competing financial interests related to the preparation or the content of the manuscript.

## Authors' contributions

LH and PIJ developed the study design and coordinated its implementation. JDN, HS, and ML participated in the interpretation and discussion of results and drafted and revised the manuscript. LH and PIJ were responsible for patient recruitment as well as data collection. LH carried out the statistical analysis. All authors read and approved the final manuscript.

## Figures and Tables

**Table 1:** Comparison of underlying disease, the effect of intrapulmonary rFVIIa therapy, and survival of DAH

Patient	Gender	Diagnosis	Pathogenesis of DAH	rFVIIa doses via BAL	Evaluation of rFVIIa effect^a^	Survival or cause of death
1	Male	Allo-HSCT^b ^(CLL)	CMV, GvHD	3	Good	Septic shock and cardiorespiratory failure
2	Male	Neurosarcoidosis	Unknown	1	Excellent	Septic shock
3	Male	AML	Unknown	1	Excellent	Survived
4	Female	Wegener's granulomatosis	Unknown	1^c^	Good	Survived
5	Female	AIDS	Unknown	2	Good	Septic shock and respiratory failure
6	Male	Allo-HSCT (AML)	Unknown	1	Excellent	Survived

^a^The hemostatic effect was statistically significant (*p *= 0.031, McNemar's test). ^b^Non-myeloablative allogeneic stem cell transplantation. ^c^One rFVIIa dose via BAL and, three days later when not intubated, subsequent three consecutive doses of rFVIIa via jet nebulizer. AML, acute myeloid leukemia; BAL, bronchoalveolar lavage; CLL, chronic lymphatic leukemia; CMV, cytomegalovirus; DAH, diffuse alveolar hemorrhage; GvHD, graft-versus-host disease; HSCT, hematopoietic stem cell transplantation; rFVIIa, human recombinant activated factor VII.

**Table 2:** Comparison of hemostatic variables and oxygenation capacity before and after rFVIIa in patients with DAH

Timing		24 hours prior to rFVIIa						24 hours after rFVIIa^a^					
Patient number		1	2	3	4	5	6	1^b^	2	3	4	5	6

Coagulation	Platelet count (10^9 ^per liter)	110	88	112	105	99	90	100	80	106	105	102	69
	APTT (seconds)	31	36	32	33	41	27	33	38	32	33	38	27
Transfusion (units)	RBCs	4	3	2	4	3	4	1	0	0	4	1	2
	FFP	4	2	2	2	2	2	0	0	0	2	0	0
	PC	2	2	2	2	2	1	0	0	0	2	0	0
Oxygenation capacity	PaO_2_/FiO_2 _ratio (mm Hg)	67	62	88	64	187	100	176	152	132	313	213	345

^a^The increase in oxygenation capacity was significant (*p *= 0.024, Wilcoxon signed paired rank test). ^b^There was a steady increase in oxygenation capacity subsequent to rFVIIa therapy (that is, the PaO_2_/FiO_2 _ratio was 176 mm Hg after one day, 137 mm Hg after one month, 136 mm Hg after two months, and finally 252 mm Hg after three months). APTT, activated partial thromboplastin time; DAH, diffuse alveolar hemorrhage; FFP, fresh frozen plasma; PaO_2_/FiO_2_, arterial oxygen pressure/inspiratory fractional oxygen content; PC, platelet concentrate; RBC, red blood cell; rFVIIa, human recombinant activated factor VII.
